# Autologous Faecal Microbiota Transplantation to Improve Outcomes of Haematopoietic Stem Cell Transplantation: Results of a Single-Centre Feasibility Study

**DOI:** 10.3390/biomedicines11123274

**Published:** 2023-12-11

**Authors:** Anna Li, Joanne M. Bowen, Imogen A. Ball, Sophie Wilson, Angelina Yong, David T. Yeung, Cindy H. Lee, Robert V. Bryant, Samuel P. Costello, Feargal J. Ryan, Hannah R. Wardill

**Affiliations:** 1 School of Biomedicine, The University of Adelaide, Adelaide, SA 5000, Australia; anna.li@adelaide.edu.au (A.L.); joanne.bowen@adelaide.edu.au (J.M.B.); 2 Supportive Oncology Research Group, Precision Cancer Medicine, The South Australian Health and Medical Research Institute, Adelaide, SA 5001, Australia; 3 Department of Gastroenterology, Basil Hetzel Institute, The Queen Elizabeth Hospital, Adelaide, SA 5011, Australia; imogen.ball@adelaide.edu.au (I.A.B.); robert.bryant@sa.gov.au (R.V.B.); scostello@biomebank.com (S.P.C.); 4 Department of Haematology, The Royal Adelaide Hospital, SA Health, Adelaide, SA 5000, Australia; sophie.wilson@sa.gov.au (S.W.); angie.yong@sa.gov.au (A.Y.); david.yeung@adelaide.edu.au (D.T.Y.); cindy.lee3@sa.gov.au (C.H.L.); 5 College of Medicine and Public Health, Flinders University, Bedford Park, SA 5042, Australia; feargal.ryan@sahmri.com; 6 Lynn Systems Immunology Group, Computational and Systems Biology Program, Precision Cancer Medicine, The South Australian Health and Medical Research Institute, Adelaide, SA 5001, Australia

**Keywords:** faecal microbiota transplantation, autologous faecal microbiota transplantation, autologous haematopoietic stem cell transplantation, haematopoietic stem cell transplantation, HSCT, bone marrow transplantation, multiple myeloma, gut microbiome, gut microbiota, supportive care, supportive oncology

## Abstract

Haematopoietic stem cell transplantation (HSCT) is a curative approach for blood cancers, yet its efficacy is undermined by a range of acute and chronic complications. In light of mounting evidence to suggest that these complications are linked to a dysbiotic gut microbiome, we aimed to evaluate the feasibility of faecal microbiota transplantation (FMT) delivered during the acute phase after HSCT. Of note, this trial opted for FMT prepared using the individual’s own stool (autologous FMT) to mitigate the risks of disease transmission from a donor stool. Adults (>18 years) with multiple myeloma were recruited from a single centre. The stool was collected prior to starting first line therapy. Patients who progressed to HSCT were offered FMT via 3 × retention enemas before day +5 (HSCT = day 0). The feasibility was determined by the recruitment rate, number and volume of enemas administered, and the retention time. Longitudinally collected stool samples were also collected to explore the influence of auto-FMT using 16S rRNA gene sequencing. *n* = 4 (2F:2M) participants received auto-FMT in 12 months. Participants received an average of 2.25 (1–3) enemas 43.67 (25–50) mL total, retained for an average of 60.78 (10–145) min. No adverse events (AEs) attributed to the FMT were identified. Although the minimum requirements were met for the volume and retention of auto-FMT, the recruitment was significantly impacted by the logistical challenges of the pretherapy stool collection. This ultimately undermined the feasibility of this trial and suggests that third party (donor) FMT should be prioritised.

## 1. Introduction

Haematopoietic stem cell transplantation (HSCT) is a curative approach used to treat a variety of blood cancers. It involves the use of high dose chemotherapy (e.g., melphalan) and, in some cases, total body irradiation (TBI), to ablate malignant blood cells, followed by haematopoietic and immune reconstitution using healthy haematopoietic stem cells collected from either the patient (autologous HSCT) or a major histocompatibility complex (MHC)-matched donor (allogeneic HSCT). While often curative, both autologous and allogeneic HSCT are associated with a range of acute and chronic complications, including infection, diarrhoea, malnutrition and graft-versus-host disease (GvHD) [1,2,3]. These complications drastically impact the quality of life, require intensive (often in-patient) supportive care and worsen the long-term prognosis [4,5,6,7].

The aetiology of HSCT-related complications is largely related to the damaging effects of the chemotherapy and TBI, which impair the homeostatic milieu of most organ systems, in particular the gastrointestinal tract. Both high dose chemotherapy and TBI cause profound injury to the mucosal lining of the gut, creating a hostile environment for the resident gut bacteria (the gut microbiome). Numerous studies have described the profound microbial changes that occur in HSCT recipients, with notable losses in commensal, short-chain fatty-acid-producing microbes such as *Blautia* that enable the expansion of enteric pathogens and pathobionts [8,9]. These detrimental changes are well documented to drive a range of acute and chronic complications, including infection (blood stream and pulmonary), diarrhoea, malnutrition and GvHD, as well as disease progression and relapse [10].

Recognising the wealth of data linking the gut microbiome with acute and chronic complications of HSCT, efforts are being made to augment the gut microbiome therapeutically. Of particular interest is faecal microbiota transplantation (FMT), a process in which faecal material containing a “healthy” microbiome collected from a healthy donor is transferred into a recipient’s gut [11]. While FMT has only been approved for the treatment of *Clostridium difficile* infection [12], it has been shown experimentally to induce remission in severe and treatment-resistant (steroid refractory) GvHD as well as eradicate multidrug resistant bacteria (MDRB) with considerable success across multiple clinical studies, albeit all relatively limited in sample size [13]. While these studies have provided important insights into the therapeutic utility of FMT for HSCT complications, there have been few attempts to use FMT to promote microbial resilience, thereby restoring commensal microbe populations lost from HSCT treatment and antibiotics and preventing the onset of complications.

In working towards an FMT strategy to support microbial resilience in HSCT, it should ideally be delivered in the acute phases following HSCT, considering it has been shown that accumulated microbial damage as early as the point of neutrophil engraftment predicts mortality [14]. This is a challenging prospect, due to immunosuppression, use of antibiotics, dietary changes and mucosal inflammation of HSCT patients, which creates a hostile environment for transplanted microbes. As such, the earliest that FMT has been studied for use in HSCT recipients is ~30 days after transplant. For example, one study conducted FMT days 8 to 27 post-engraftment, including a patient receiving FMT 49 days after HSCT [15].

Here, we aimed to investigate the feasibility of an early intervention FMT in autologous HSCT recipients, delivered in the acute stages of microbial damage. In addition, we opted for the use of autologous FMT preparation (i.e., FMT prepared from patient’s own stool) to recognise the high interindividual variation in the microbiome. Although there have not been any studies to directly compare donor vs. autologous FMT, the microbial interactions are important for FMT engraftment, where new microbial strains from a donor have a higher likelihood of engrafting if the recipient already possesses that species [16]. This is especially important when considering that the gut microbiome is as “unique as a fingerprint”, being shaped by evolution and the environment, diet and diseases experienced through life [17,18,19]. Recognising that microbial interactions with mucosal innate immune cells are critical for developing immune tolerance [20], the symbiosis between the host and their microbiome may facilitate engraftment and thus the efficacy of FMT. As such, autologous FMT may be better positioned to promote engraftment. This method also minimises the risk of donor-to-recipient disease transmission, which has been documented in other FMT settings [21].

## 2. Materials and Methods

### 2.1. Study Recruitment and Eligibility

This study was an open-label, non-randomised feasibility study performed in 2021 at the Royal Adelaide Hospital. All study protocols were approved by the Central Adelaide Local Health Network Human Research Ethics Committee (HREC/17/RAH/533), in accordance with the Declaration of Helsinki.

Participants were deemed eligible to participate if they were adult (aged > 18 years), were cytotoxic treatment naive and diagnosed with a haematological malignancy that was likely to require auto-HSCT. Participants were excluded if they had intestinal symptoms at the time of recruitment, a medical history of a gastrointestinal disorder including inflammatory bowel disease and irritable bowel syndrome, had previous colonic surgery (excluding colonoscopy), were pregnant, were unable to provide informed consent or were unable to provide a baseline (pretherapy) stool sample. All the participants were identified at the point of diagnosis, at which point they provided written and informed consent to donate a fresh stool sample, which was processed in accordance with international guidelines [11]. Once the participants were scheduled to receive HSCT, FMT was offered to those participants who had a viable baseline (pretherapy) stool sample (see Section 2.2). The eligible participants then provided an updated information sheet and consent form, and were free to withdraw at any time without reason.

### 2.2. FMT Preparation and Administration

The baseline stool was collected from participants at diagnosis, prior to starting first line therapy, and used to prepare the autologous FMT according to international standards [11]. The participants were instructed to pass urine before defecating into a stool collection bag placed over the toilet, which was sealed with a zip tie and stored at 4 °C until collection. A maximum of 6 h was permitted between defecation and collection. To prepare the FMT, the stool was homogenised under anaerobic conditions using a Stomacher4000 with clinical-grade saline and glycerol at a ratio of 25–65–10% (saline-stool-glycerol). The FMT preparations were stored at −80 °C in 50 mL enema syringes until administration. An aliquot of the FMT product was sent for routine stool screening (performed at SA Pathology) to identify *Clostridium difficile* toxin, ova, cysts and parasites (CAD/MCS/OCP/Viral PCR–stool microscopy, culture and sensitivity test). A positive stool screening rendered the FMT not viable for use and participants were therefore ineligible. FMT was only performed if the participants were afebrile (oral temperature < 37.5 °C), had ANC > 1.0 × 10^9^/L, platelets > 5.0 × 10^10^/L and had no evidence of rectal bleeding.

**Figure 1:**
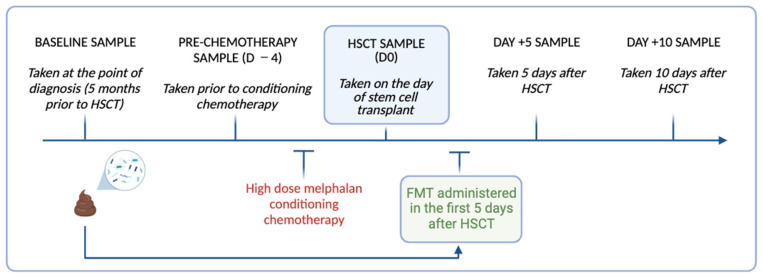
Timeline of longitudinal stool collection and FMT delivery relative to high dose melphalan conditioning chemotherapy and HSCT in the study participants. Image created using Biorender.

FMT was administered to N = 4 autologous HSCT recipients via 3 × 50 mL retention enema (FMT1.1, 1.2, 1.3) administered within five days of HSCT (Figure 1). Loperamide (2 mg) was given orally 2 h prior to facilitate retention. Participants were directed to retain the enema for a minimum of 30 min. All participants received standard supportive care. Procedural feasibility was determined by an uptake of a minimum of 50 g of FMT and the number, volume and retention time of the enemas delivered.

### 2.3. Clinical Assessments and Adverse Events

In addition to routine clinical assessments, body weight, stool consistency (Bristol Stool Chart), stool frequency and abdominal pain were assessed before conditioning chemotherapy (Day −4), day of HSCT (Day 0), Day +5 and Day +10. Any adverse events were evaluated using the Common Terminology Criteria for Adverse Events (CTCAE), v4.0.

### 2.4. Stool Collection and 16S rRNA Gene Sequencing

Repeated stool samples were collected from the participants at the time of FMT preparation (S0), before conditioning chemotherapy (D −4), the day of HSCT (D0), and day +5 (D +5) and day +10 (D +10) for microbial analysis (Figure 1). Stool samples (~1 g) were collected by the participants using self-collection Zymo research DNA/RNA shield faecal collection tubes (Zymo Research, Irvine, CA, USA Catalogue no./ID: R1101) and stored at −80 °C until processing. On the day of processing, the stool samples were thawed to room temperature and DNA extracted using the Dneasy Powerlyzer PowerSoil Kit (Qiagen Catalogue. No./ID: 47016) as per the manufacturer’s instructions. The DNA concentration was quantified using a Qubit 2.0 Fluorometer (Thermo Fisher Scientific, Adelaide, SA, Australia). The samples were sent to the South Australian Genomics Centre (SAGC, Adelaide, Australia) for 16S rRNA gene sequencing, performed via Illumina Miseq (Illumina, San Diego, CA, USA) using primers targeting the hypervariable V3-V4 region [primers: 314F: 5′-CCTACGGGNGGCWGCAG-3′ 806R: 5′-GACTACHVGGGTATCTAATCC-3′]. The raw sequence data generated as part of this research has been deposited in the Sequence Read Archive under BioProject ID: PRJNA1045367.

### 2.5. Bioinformatics

16S bioinformatics (clustering and analyses) were performed using the Qiagen CLC genomics platform (Version 12.0). The paired-end data were merged with a minimum distance of 200 and maximum of 500. This was trimmed using the Trim reads tool with quality limited to 0.05 and the maximum number of ambiguities set to two. Operational taxonomic unit (OTU) picking was performed with chimera filtering using the Silva 16S and 18S v132 99% database. The OTUs were aligned with MUSCLE and alpha diversity determined through Shannon’s diversity.

### 2.6. Statistical Analysis

The statistical analysis was performed in the Qiagen CLC Genomics Workbench v22.0.2 and GraphPad Prism v9.3.1. Alpha diversity and OTUs were assessed using a one-way repeated measures analysis of variance (ANOVA) with comparison between all timepoints. For the comparison of microbial similarity between S0 and day 10, Spearman’s correlation between samples was calculated and plotted as a heatmap in a GraphPad Prism from relative abundance of OTUs at the genus level.

For further insight into the effect of auto-FMT, the OTU relative abundances were summed by sample origin (OTUs that are detected at both S0 and D10; detected at D10 only; and detected at S0) using R statistical software V4.3.0. OTU detection was defined as greater than or equal to a relative abundance of 0.1%

## 3. Results

### 3.1. Feasibility of Auto-FMT in In-Patient HSCT Recipients

FMT was performed in N = 4 people (2F/2M) receiving auto-FMT. No participants were excluded due to positive stool screening. All participants received 50 g of FMT. An average of two enemas were administered to the participants, with an average administration volume of 44.13 mL and retention time of 63.18 min (Supplementary Table S1). Patient 4 received only one FMT enema (FMT1.1), which was provided in the outpatient clinic, and did not want to proceed with the remaining FMTs due to difficulties attending the outpatient clinic. For body weight, stool frequency and stool consistency across the study time points, see Supplementary Table S2. Due to the small sample size, no grouped analyses were performed. No adverse events attributable to the FMT were identified.

### 3.2. Longitudinal Gut Microbiome Changes

**Figure 2:**
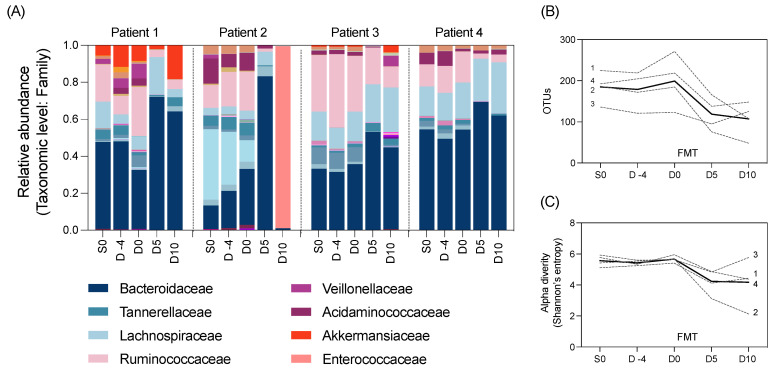
Microbial taxa at the family level by (**A**) relative abundance, as well as (**B**) richness and (**C**) alpha diversity of the gut microbiota in N = 4 participants pre- and post-FMT intervention (unbroken line indicates the average across patients). Normality determined using the Shapiro–Wilk test. Data analysed using repeated measures one-way ANOVA. No significant differences were identified in any microbial outcome measures between time points.

We also aimed to explore changes to the gut microbiome throughout the study using 16S rRNA gene sequencing of the longitudinal stool samples. The composition of the gut microbiota in all participants was largely composed of the *Bacteroidaecceae* family (Figure 2A). All participants had a marked decrease in alpha diversity and microbial richness after HSCT (Day 0 to 5) (Figure 2B,C).

**Figure 3:**
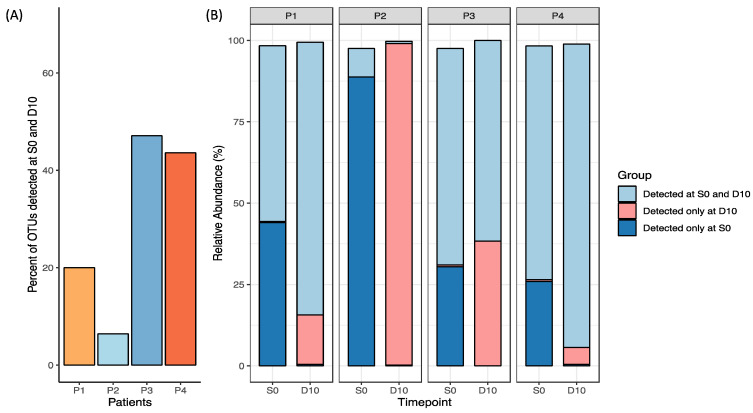
Relative OTU abundance in each participant’s S0 and D10 sample plotted as (**A**) percentage of OTUs detected in both S0 and D10 (engraftment), and (**B**) groups detected at S0 and D10, D10 only and S0 only relative to one another (excluding taxa < 0.1% abundance).

Measures of relative OTUs uniquely present in both S0 and D10 were used to measure the auto-FMT uptake. For Patient 1, ~20% of OTUs detected were in this group, while for Patient 4 this comprised ~40% of the OTUs detected (Figure 3). Notably, Patient 3 had the greatest proportion of OTUs detected in both S0 and D10; however, this was observed alongside a rise in OTUs detected only in D10, indicating microbial changes attributable to HSCT treatment was contributing to its dissimilarity to baseline. Due to a low N, no definitive trends or conclusions can be determined as a result of the FMT. This analysis was supplemented by analysis of the degree of similarity between the baseline (S0) stool sample used to prepare the FMT and day 10 stool samples collected after FMT (Supplementary Figure S1).

## 4. Discussion

Patterns of microbiota disruption in HSCT recipients have become increasingly recognised to contribute to HSCT complications such as sepsis, diarrhoea, malnutrition, and graft-versus-host disease. FMT has shown considerable success at restoring a healthy microbiota composition with demonstrated efficacy in some of these chronic HSCT complications [13]. However, FMT’s success has been limited by a lack of understanding of its potential utility and efficacy when delivered in the acute phases of HSCT. We therefore aimed to test the feasibility of an early intervention FMT, prepared using autologous stool, in a cohort of HSCT recipients. Ultimately, this protocol was not feasible to implement in our institute, largely due to the logistical challenges of pre-treatment (autologous) stool collection. Hence, we were unable to effectively draw conclusions on the efficacy and safety of autologous FMT. Nonetheless, our study provided exploratory insight into the considerations for acutely delivered FMT.

Although auto-FMT has scientific rationale in its potential to reconstitute the patient’s unique healthy microbiome, as well as reduce the risk of donor-to-recipient disease transmission, collection of the pretherapy stool was a major barrier to recruitment and feasibility to our approach. In particular, we implemented a stringent approach to baseline stool collection, where faecal samples are taken before any cytotoxic therapy has commenced. As a result, we were only able to perform FMT in a very small number of participants and thus our results must be interpreted with extreme caution. This contrasts with the approach used by Taur and colleagues, who prepared auto-FMT using stool collected from patients after first line therapy but before transplant conditioning [15]. They did however incorporate methods to exclude samples with a low microbial viability. Although this presents as a potentially more feasible approach, with no concrete evidence to suggest that auto-FMT is superior to donor FMT it remains unclear if the focus on auto-FMT is worthy of the logistical burden. This does however raise an important question that remains unanswered in the literature, which is, if auto-FMT is feasible, should it be prioritised over donor FMT? This is also of interest in the setting of allogeneic HSCT, where the immune system is no longer that of the recipient but instead, a donor. In this context, it is of interest to consider if FMT prepared using the stem cell donor’s stool would be optimal to enhance immune-microbiome stability.

In addition to autologous preparation, a novel aspect of our approach was the timing of the FMT, which was delivered early after HSCT to offer prophylaxis against dysbiosis induced complications. When considering early FMT delivery, namely its ability to colonise the gut to induce a beneficial effect, the hostility of the gastrointestinal microenvironment is critical. Mucositis and other factors (e.g., antibiotics, dietary changes) undoubtedly create a hostile environment for microbes (endogenous and exogenously delivered). Thus, the administration of FMT during “peak” gastrointestinal hostility is a challenging paradox, holding superior potential compared to therapeutic use, yet being inherently complex. Our study, albeit with a small sample, showed that the capacity to deliver FMT by enema acutely after HSCT is potentially feasible, with the average volume and retention time meeting the requirements of 50mL and 30 min. Of course, it must be noted that this was only performed with a small number of participants, and thus this conclusion must be confirmed in a larger study. Similarly, we cannot ignore the fact that the FMT delivered in the outpatient setting required placed a significant burden on the participant, impacting their ability to receive all three enemas. Moving forward, identifying strategies to overcome the logistical challenges of enema-delivered FMT, such as using encapsulated FMT instead, should be prioritised. Encapsulated FMT may also offer a longer duration of microbial input through repeated longitudinal dosing, thus optimising colonisation in the face of numerous co-occurring microbial insults that occur in HSCT recipients (e.g., antibiotics). In light of advances in FMT formulations, encapsulated FMT, which has shown efficacy for other FMT indications e.g., *Clostridium difficile* infections [22], may be the most appropriate modality in this unique clinical setting.

While we provide some novel insight into the feasibility of early intervention auto-FMT, it must be viewed in light of the study’s limitations. First, and most critically, the low sample size cannot be ignored. This ultimately highlights the logistical challenges of our protocol, which we believe is largely dictated by the collection of autologous stool and the reliance on enema delivery. With the low sample size, our ability to draw robust conclusions is severely impacted, both with respect to the efficacy and safety of the intervention, as well as its impact on the gut microbiota. As such, all microbial data must be considered as strictly exploratory. Second, our study did not include a placebo or control group. Thus, while we observed no serious adverse events attributed to FMT in our study, more rigorous safety assessments are needed in a larger patient population where the FMT is delivered close to or during periods of immunosuppression. This, in addition to being a single-centre study with a high degree of outpatient HSCT service, also resulted in an underpowered study that may not necessarily reflect other settings. While these points are critical in the interpretation of our results, we felt it necessary to disseminate these findings to ensure future efforts to support the gut microbiota in HSCT recipients are designed appropriately to ensure feasibility and, ultimately, improved outcomes for HSCT recipients.

## 5. Conclusions

Despite the scientific rationale and safety advantages of auto-FMT, our results highlight the major logistical challenges in implementing an auto-FMT protocol. It is likely that for a protocol comparable to ours to be successful, an enormous infrastructure will be required to facilitate pretherapy autologous stool collection and processing. Without evidence to suggest auto-FMT is superior to donor FMT, we suggest future efforts focus on the ability of early intervention, encapsulated donor FMT to promote microbial resilience and improve the outcomes of HSCT. The authors recognise that the number of patients is very small, but still feel that the scientific community should be informed of these findings.

## Data Availability

The data presented in this study are available on request from the corresponding author.

## Supplementary Materials

The following supporting information can be downloaded at: https://www.mdpi.com/article/10.3390/biomedicines11123274/s1, Table S1: Clinical parameters and characteristics of N = 4 participants receiving autologous FMT; Table S2: Clinical assessments of N = 4 participants receiving autologous FMT; Figure S1: Spearman’s correlation of longitudinal samples against the baseline sample of each patient as a graph (A) and as heatmaps for patients (B) 4 and (C) 1, (D) 2 and (E) 3. Data generated from relative abundance of OTUs at the genus level. ** indicates *p* < 0.01, * indicates *p* < 0.05 detected by one-way ANOVA against S0.
