# Supplementary material for: Autologous Faecal Microbiota Transplantation to Improve Outcomes of Haematopoietic Stem Cell Transplantation: Results of a Single-Centre Feasibility Study
Source: Biomedicines. 2023 Dec 11;11(12):3274. doi: 10.3390/biomedicines11123274 (PMC10741751; doi:10.3390/biomedicines11123274)
Supplement: Supplementary file 1 [file biomedicines-11-03274-s001.zip › biomedicines-2732552-supplementary.pdf]

Supplementary tables and figures

Table S1. Clinical parameters and characteristics of N=4 participants receiving autologous FMT

	**Patient 1**	**Patient 2**	**Patient 3**	**Patient 4**
**Age (years) at time of HSCT**	47	53	65	34
**Sex**	Male	Female	Male	Female
**Diagnosis**	IgG K Multiple Myeloma	Kappa light chain Multiple Myeloma	IgG K Multiple Myeloma	IgA K Multiple Myeloma
**Conditioning**	HDM 140mg/m^2	HDM 200mg/m^2	HDM 200mg/m^2	HDM 200mg/m^2
**Prophylactic and empirical antibiotics, antivirals, antifungals**	Norfloxacin 400mg BD, Famciclovir 250mg BD, Fluconazole 200mg OD Cefepime (D+9)	Trimethoprim/Sulfamethoxazole 160/800mg Famciclovir 500mg BD Tazocin (D+7)	Trimethoprim /Sulfamethoxazole 160/800mg BD Famciclovir 500mg BD Entecavir 0.5mg Norfloxacin, Fluconazole Azithromycin (D+8)	Trimethoprim /Sulfamethoxazole 160/800mg BD Famciclovir 500mg BD Norfloxacin 400mg BD Fluconazole 400mg (continued until D+10)
**Body weight (kg) on day of FMT1.1**	85	74	81	65
**Number of enemas given and days relative to HSCT**	3 (+2 to +4)	3 (+2 to +4)	2 (+3 to +4) *FMT1.3 not given due to low ANC*	1 (+1) *FMT1.2 and 1.3 not given due to participant preference*
**Volume as mean ± SEM (mL)**	33.3 ± 14.4	50.0 ± 0.0	51.5 ± 2.1	40.0 ± 0.0
**Retention time as mean ± SEM (min)**	97.3 ± 47.0	63.7 ± 16.2	14.0 ± 5.7	36.0 ± 0.0
**Adverse events**	N/A	N/A	Campylobacter gastroenteritis infection on D+8 (not traced to FMT), and engraftment syndrome (after day 10)	N/A

Table S2. Clinical assessments of N=4 participants receiving autologous FMT

	**Patient 1**				**Patient 2**				**Patient 3**				**Patient 4**			
	Day -4	Day 0	Day 5	Day 10	Day -4	Day 0	Day 5	Day 10	Day -4	Day 0	Day 5	Day 10	Day -4	Day 0	Day 5	Day 10
BW (kg)	85*	91.5	86.3	88	74	74.8	72.2	73.3	81*	83	79.9	80.5	65*	66.3	63.4	64.1
Stool consistency (BRS)	4	5	5	*7*	2.5	1	4	6.5	2.5	1	4	6.5	3	1	5	1
Stools/day	1	1	1	7	1	1	3	2	1	1	3	2	4	2	4	2
Rectal bleeding (Y/N)	N	N	N	N	N	N	N	N	N	N	N	N	N	N	N	N
Abdominal pain (Y/N)	N	N	N	N	N	Y	N	N	N	N	N	Y	N	N	N	N

**indicates at-home (patient reported) measurement*

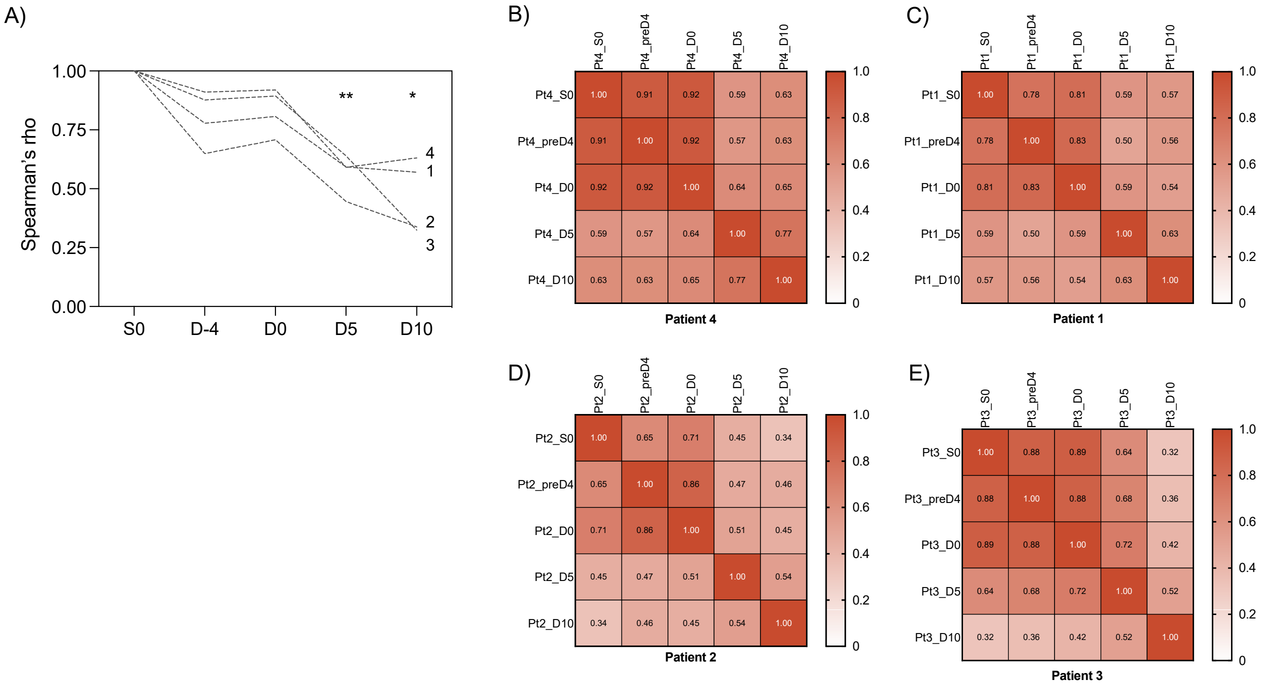

Figure S1. Spearman's correlation of longitudinal samples against the baseline sample of each patient as a graph (**A**) and as heatmaps for patients **(B)** 4 and **(C)** 1, **(D)** 2 and **(E)** 3. Data generated from relative abundance of OTUs at the genus level. ** indicates P<0.01, * indicates P<0.05 detected by one-way ANOVA against S0.
